# Discovering the mechanism and involvement of the methylation of cyclin-dependent kinase inhibitor 2A (CDKN2A) gene and its special locus region in gastric cancer

**DOI:** 10.1080/21655979.2021.1887646

**Published:** 2021-04-26

**Authors:** Jiye Xu, Ning Li, Wenying Deng, Suxia Luo

**Affiliations:** a Department of Oncology, Henan Cancer Hospital, the Afﬁliated Cancer Hospital of Zhengzhou University, Zhengzhou, China; b Department of Medical Oncology, Zhoukou Central Hospital, Zhoukou, Henan, China

**Keywords:** CDKN2A, methylation, gastric cancer, GSEA, TCGA

## Abstract

Cyclin-dependent kinase inhibitor 2A (CDKN2A) gene methylation has been paramount in the development of malignant masses. The purpose of the conducted research was to evaluate the mechanism and involvement of methylation in regards to the CDKN2A gene and the specific locus region in gastric cancer (GC) with comprehensive statistical analysis utilizing statistics acquired from The Cancer Genome Atlas (TCGA) database. Gene Set Enrichment Analysis (GSEA) revealed that the level of CDKN2A gene methylation and its locus in GC tissues was increased compared to para-cancerous tissues. In multivariate analysis, low methylation of CDKN2A gene, cg03079681, cg04026675, cg07562918, and cg13601799 locus were independently linked to better OS. In addition, the methylation of CDKN2A gene, cg00718440, cg03079681, cg04026675, cg07562918, cg10848754, cg14069088 and cg14430974 locus were negative correlated with CDKN2A gene expression. Meanwhile, the methylation of cg12840719 locus was positively correlated with CDKN2A gene expression. GSEA showed that hallmark_kras_signaling_dn, hallmark_myogenesis, and hallmark_epithelial_mesenchymal_transition pathways were enriched in the CDKN2A gene hypermethylation phenotype. Taken together, the low methylation of CDKN2A gene, cg03079681, cg04026675, cg07562918, and cg13601799 locus indicated a better prognosis in GC. The methylation levels of cg14069088 were most negatively correlated with CDKN2A gene expression. Hallmark_kras_signaling_dn, Hallmark_myogenesis, and hallmark_epithelial_mesenchymal_transition pathways might be important in the regulation of CDKN2A gene hypermethylation.

## Graphical abstract

**Figure:**
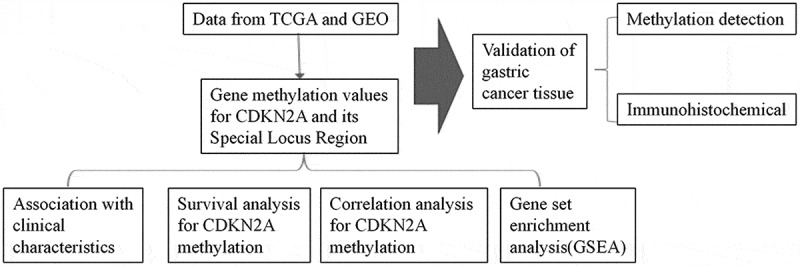

## Introduction

Gastric cancer (GC) is considered to be the fourth major prevalent malignancy and the third cause of cancer-associated deaths worldwide [1]. In East Asia, one prime source of cancer-related mortalities is GC [2]. Diagnostic and treatment for early detected GC have seen rapid growth in recent times [3]. Considering this, treatments for late stages remain at a low. Secondary therapy which involves chemoradiotherapy post-surgery has proven to yield unsatisfactory outcomes. This along with the use of paclitaxel and ramucirumab, which is an antibody VEGFR-2 antagonist, the general overall survival (OS) persists at under 2 years [4,5]. It has been known that aberrant DNA methylation alters the tumor microenvironment and can be used to diagnose disease and estimate prognosis. Research has shown that abnormal DNA methylation of individual genes could be crucial in GC pathogenesis and development [6]. There is increasing evidence suggest that measuring DNA methylation is important for early disease screening, evaluating treatment efficacy, and estimate prognosis in GC patients [7].

Cyclin-dependent kinase inhibitor 2A (CDKN2A) encodes and results in a production of p16 genes that are involved with a range of cellular pathways which consists of suppression of the cancer cell cycle, cell senescence, apoptosis, differentiation, and DNA repair [8,9]. Recent studies conducted on colon cancer suggested that both the CDKN2A gene methylation and the lack of CDKN2A expression were not related to colorectal cancer-specific passings. Patients who possess the CDKN2A methylation gene, as well as the drop in CDKN2A expression, suffered from a decreased OS rate [10].

At present, there have been reports on the levels of methylation in regards to CDKN2A promoters in GC [11]. However, the methylation of some genes is not necessarily the key methylation region of genes, and the relationship with prognosis is not comprehensive. In addition, most studies that examine these associations were case series or basic research with a limited number of samples. Larger studies with comprehensive bioinformatics are needed to elucidate the clinical and biological significance of CDKN2A gene methylation in GC.

Therefore, in this study, for the first time, we analyzed the methylation level of the CDKN2A gene region and the methylation of each site in GC through bioinformatics and its relationship with the prognosis of GC.

## Methods

### Data resource and description

The preprocessed three-gene methylation and gene expression data, as well as the associated clinical data of patients diagnosed with primary GC, were transferred from the TCGA data portal in June 2020. There were a total of 406 primary gastric tumor patients with disease type of ‘adenomas and adenocarcinomas’ in the Cancer Genome Atlas (TCGA) data. TCGA RNAseq data contain 373 samples which including 343 primary GC and 30 para-carcinoma samples. TCGA methylation data obtained from the Infinium Human Methylation 450 array contain 362 samples which including 360 primary GC and 2 para-carcinoma samples and the Infinium Human Methylation 27 array contains 71 samples which including 46 primary GC and 25 para-carcinoma samples. One GSE30601 methylation data from the Infinium Human Methylation 27 array contain 203 primary GC and 94 para-carcinoma specimens were gained from the Gene Expression Omnibus dataset (GEO, https://www.ncbi.nlm.nih.gov/geo/). Platforms and specimens we used in this study are summarized in Table 1.

**Table 1.** Details of TCGA and GEO data included in this analysis

Data	Normal	Tumor
TCGA mRNA data	30	343
TCGA HumanMethylation27 assay	25	46
TCGA HumanMethylation450 assay	2	360
GSE30601 HumanMethylation27 assay	94	203

## Survival curve and prognosis analysis

Kaplan–Meier was conducted for examining the OS rates based on different gene expression and methylation levels by using R software (v.3.5.2). Cutoff levels were set at median value with applied Log-Rank. The Cox proportional hazard regression model was used to carry out the univariate survival analysis with OS time of 5 years to analyze prognostic utilities. The comparison of CDKN2A gene methylation on survival in addition to supplementary characteristics was done via the Multivariate Cox examination.

## Gene set enrichment analysis (GSEA)

TCGA gastric cancer database, cases were separated into 2 as per the median methylation value of CDKN2A. Detection of sets of genes that were enhanced in the gene rank was carried out by GSEA. 1,000 permutations were conducted under the selection of interpreted gene sets of h.all.v7.0.symbols.gmt in the Molecular Signatures Database (MSigDB) in GSEA version 3.0, Collapsed datasets to gene symbols were regarded as ‘false’ while ‘phenotype’ represents permutation type. GSEA was done and the cut-of was: false discovery rate (FDR) q > 0.25 and nominal P < 0.05.

## Human tissue collection

From January 2015 to June 2016, 62 pairs of paraffin-embedded tissue samples were collected at Zhoukou central hospital after GC surgery and each pair consisted of primary cancer tissue and adjacent non-cancerous tissue. Associated clinical information was collected. The histological diagnosis was confirmed by an independent pathologist. White tissue slices were cut in the adjacent position of the embedded tissue, and immunohistochemical detection and methylation detection were performed. Ethics Committee of Zhoukou central hospital has authorized the protocols relating to this research.

## Immunohistochemical assay

Glass slides of Formalin-fixed paraffin-embedded (FFPE) sections were used for immunohistochemistry conducted under standard protocols. Specifically, the sections were first resolved in xylene, before hydration with graded ethanol and washed under tap water and distilled water. A methanol solution consisting of 0.3% hydrogen peroxide was used to inhibit endogenous peroxidase activity for 30 minutes. Sections were incubated in a 10 mol/L citrate buffer solution (pH: 6.0) before it was boiled for 10–15 minutes to obtain adequate antigen repair. Tissue sections were then sealed at room temperature in 10% normal goat serum for 1 hr before an all-night incubation 4°C with anti-CDKN2A/p16INK4a antibody (Abcam, ab54210; 1:200; Cambridge, UK). Rinsing with PBS was done prior to the application of the secondary antibody (Abcam, ab7010; 1:500) and the slides were incubated for 1 h at 37°C. Finally, tissue sections were revealed for 5 minutes to diaminobenidine peroxidase substrate before staining in hematoxylin and eosin. Image inspection was performed by employing Image-Pro Plus 6.0 software. Each immunohistochemical staining was repeated 3 times independently.

## Methylation-specific PCR (MS-PCR)

A DNA extraction kit (Tiangen biotech, Beijing, China) was manipulated to extract the DNA from the embedded tissue following standard protocol. Subsequent methylation-specific PCR (MS-PCR) was performed under the DNA bisulfite conversion kit and MS-PCR kit with full compliance to manufacturer instructions. The MethPrimer online tools (www.urogene.org/methprimer) were applied to design methylation-specific PCR primers. Each group of primers was used for independent PCR amplification under the following conditions: 94°C for 5 min, then 40 cycles of 94°C for 45 s, 56°C for 45 s, and 72°C for 45 s, and the final extension step was 72°C for 10 min. 1.8% agarose gel electrophoresis was conducted to examine the gained PCR products and the number of methylation in the samples was counted.

## Statistical analysis

Differences in mean methylation between GC and normal tissues were evaluated with dependent Wilcoxon signed-rank tests. CDKN2A gene methylation expression in clinical-pathological features was studied accompanied by the use of Wilcoxon signed-rank test and logistic regression assay. Pearson correlation assay was incorporated to determine the association between CDKN2A gene methylation and corresponding CDKN2A expression levels obtained from TCGA expression data (filtering conditions cor < 0.2, P < 0.05). The Kaplan-Meier analysis was conducted to examine the OS rates based on different gene expression and methylation levels by using R software (v.3.5.2). Cutoff levels were at median value along with the application of Log-Rank Univariate survival analysis was done in collaboration with the Cox proportional hazard regression model including a OS time (5-year) to evaluate prognostic value. The inﬂuence of CDKN2A gene methylation on survival was weighed up alongside the Multivariate Cox analysis with the addition of other clinical characteristics. Only P values below 0.05 were noted to be analytically weighed.

## Results

### Patient characteristics

First, we analyzed the clinical information of GC patients in the TCGA database. Table 2 illustrates a total of 406 primary GC patients’ clinical data gained from TCGA in June 2020. There were 36.9% female and the average age diagnosed was 67 years old (30–90). The histologic grade (G) of gastric cancer included G1 (2.5%), G2 (37.3%), and G3 (60.2%). Tumor 1 (T1) was found in 23 patients (5.8%), T2 in 85 (21.5%), T3 in 185 (46.7%) and T4 in 103 (26.0%). A total of 267 patients had lymph node metastases and 27 patients had distant metastases. Stage I disease were present in 56 patients (14.7%), stage II in 118 (31.1%), stage III in 167 (43.9%), and stage IV in 39 (10.3%). The Median follow-up period till the last contact was 385 days (range, 0–3720 days).

**Table 2.** Characteristics of TCGA gastric cancer patients

Parameters	Total number, n (%)
Total	435
Age (years)	
≤60	141 (32.4)
>60	294 (67.6)
Gender	
Male	279 (64.1)
Female	156 (35.9)
Histologic grade	
G1	12 (2.8)
G2	152 (35.7)
G3	262 (61.5)
Tumor	
T1	21 (4.9)
T2	89 (20.9)
T3	196 (46.1)
T4	119 (28.0)
Lymph nodes	
Negative	128 (30.8)
Positive	288 (69.2)
Metastasis	
Negative	385 (92.8)
Positive	30 (7.2)
Stage	
I	55 (13.5)
II	129 (31.6)
III	181 (44.4)
IV	43 (10.5)

## Relationship between CDKN2A gene methylation and expression

**Figure 1.**
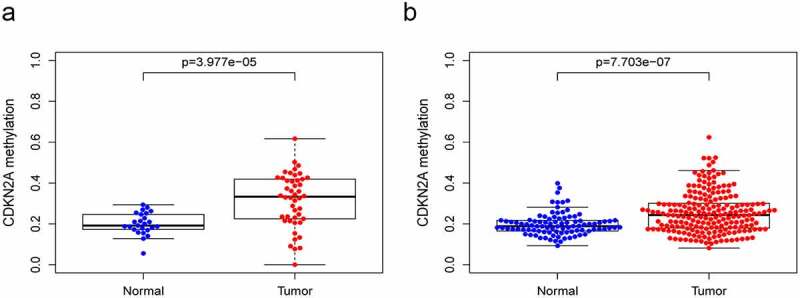
The methylation level of CDKN2A gene grew in GC tissues when viewed in contrast with normal gastric tissues. (a) TCGA datasets. (b) GSE30601 datasets

We determined the correlation between CDKN2A gene methylation and its locus and CDKN2A gene expression in GC. A total of 71 samples with 46 primary GC and 25 para-carcinoma samples obtained from TCGA HumanMethylation27 and 297 samples including 203 primary GC and 94 para-carcinoma samples obtained from GSE30601 HumanMethylation27 were analyzed. Our data suggested in both TCGA datasets (Figure 1(a)) and GSE30601 datasets (Figure 1(b)), the methylation level of CDKN2A gene rocketed in GC tissue in contrast with normal samples (P = 3.977e-05 and P = 7.703e-07).

In addition, a total of 362 samples including 360 primary GC and 2 normal samples obtained from TCGA HumanMethylation450 were analyzed along with 9 methylation sites: cg00718440, cg03079681, cg04026675, cg07562918, cg10848754, cg12840719, cg13601799, cg14069088, and cg14430974.

**Figure 2.**
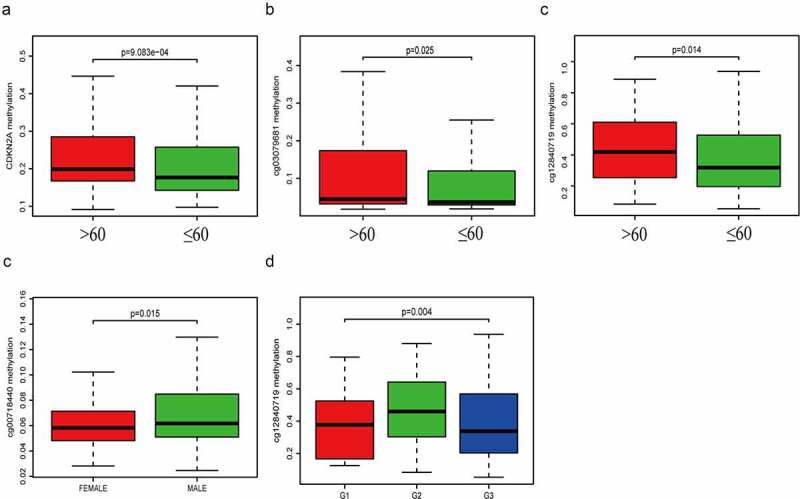
Correlation of CDKN2A gene methylation and expression on TCGA data. (a) CDKN2A gene methylation with CDKN2A expression. (b) cg00718440 methylation with CDKN2A expression. (c) cg03079681 methylation with CDKN2A expression. (d) cg04026675 methylation with CDKN2A expression. (e) cg07562918 methylation with CDKN2A expression. (f) cg10848754 methylation with CDKN2A expression. (g) cg14069088 methylation with CDKN2A expression. (h) cg14430974 methylation with CDKN2A expression. (i) cg12840719 methylation with CDKN2A expression

Furthermore, the results of Figure 2 suggested that quantities of CDKN2A gene methylation were in inverse relationship with CDKN2A gene expression (rs = −0.224, P = 7.905e-05). Similarly, the methylation levels of cg00718440, cg03079681, cg04026675, cg07562918, cg10848754, cg14069088 and cg14430974 also negatively correlated with CDKN2A gene expression (rs = −0.209, P = 2.393e-04; rs = −0.406, P = 1.393e-13; rs = −0.207, P = 2.672e-04; rs = −0.407, P = 1.161e-13; rs = −0.253,P = 7.398e-06; rs = −0.567, P = 2.172e-27; rs = −0.251, P = 9.143e-06, respectively) (Figure 2(a-h)). However, the methylation of cg12840719 was positively correlated with CDKN2A gene expression (rs = 0.483, P = 2.667e-19) (Figure 2(i)).

## Association between CDKN2A gene methylation and clinicopathologic parameters

**Figure 3.**
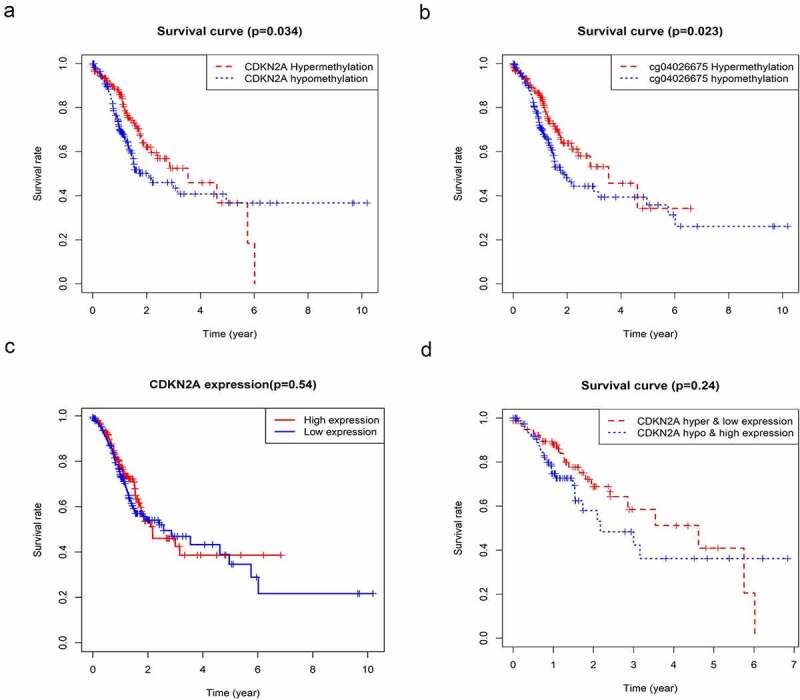
The relationship linking CDKN2A gene and each locus methylation with clinicopathologic parameters. (a) CDKN2A gene methylation with age. (b) cg03079681 methylation with age. (c) cg12840719 methylation with age. (d) cg00718440 methylation with gender. (e) cg12840719 methylation with tumor grade

The average general CDKN2A gene DNA methylation level and methylation levels at each locus were analyzed with corresponding clinicopathologic parameters (age, gender, grade, TNM stage). Our data suggested that CDKN2A gene (Figure 3(a)), cg03079681 locus (Figure 3(b)) and cg12840719 locus (Figure 3(c)) methylation were significantly increased in elderly patients (>60 years old) (P = 9.083e-04, 0.025 and 0.014). The methylation level of cg00718440 (Figure 3(d)) were remarkably elevated when comparing male and female patients (P = 0.015). Levels of methylation of cg12840719 (Figure 3(e)) were much greater in individuals of G2 grade (P = 0.004). Other methylation sites were not statistically significant, and the data were not shown.

## Association between CDKN2A gene methylation and survival rate

**Figure 4.**
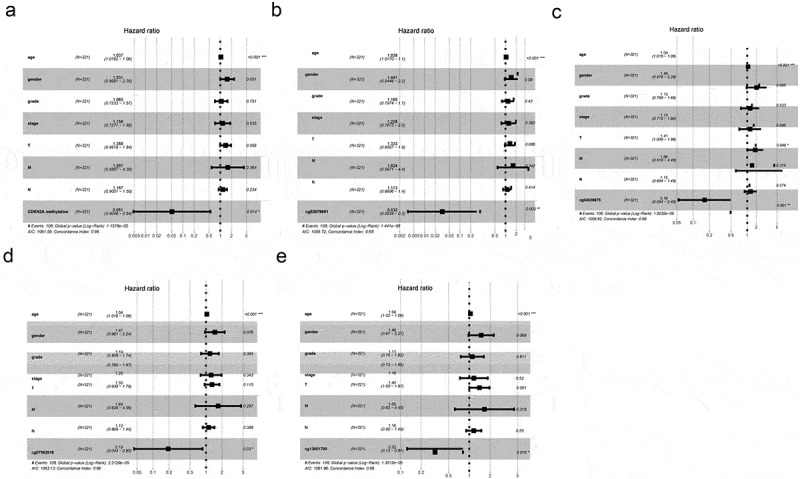
The association between CDKN2A gene methylation and expression with OS based on TCGA data. (a) Correlation of CDKN2A gene methylation with OS. (b) Correlation of cg04026675 methylation with OS. (c) Correlation of CDKN2A expression with OS. (d) Correlation of CDKN2A gene high methylation and low expression with OS. Hyper & low expression, hypermethylation, and low expression; hypo & high expression, hypomethylation, and high expression. OS

To further determine the relationship between CDKN2A gene methylation and its locus and the clinical prognosis of GC. A summary of 360 primary GC samples obtained from TCGA Humanmethylation450 was used for the Kaplan-Meier survival study and the result demonstrated that GC patients that have hypermethylation of CDKN2A gene and cg04026675 had a better prognosis at the 5-year follow-up (Figure 4(a,b)). However, the survival rate reversed in the last 5 years. (Figure 4(c,d)) showed that the above observation followed a similar pattern but did not reach a significant difference when using primary GC samples obtained from TCGA.

**Figure 5.**
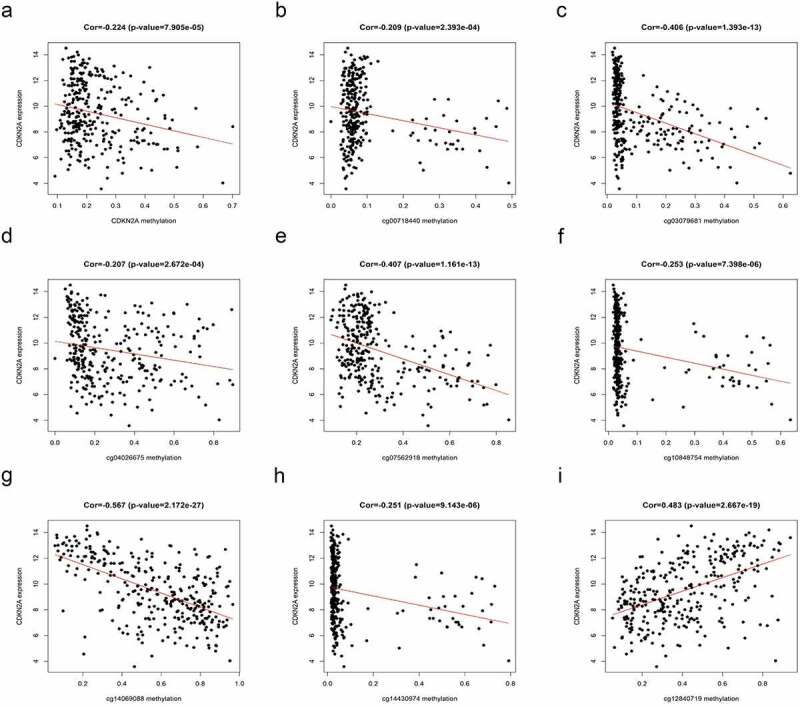
OS rate by prespecified patient subgroups defined by baseline characteristics. (a) CDKN2A gene methylation and other clinicopathologic variables; (b) cg03079681 methylation and other clinicopathologic variables. (c) cg04026675 methylation and other clinicopathologic variables. (d) cg07562918 methylation and other clinicopathologic variables. (e) cg13601799 methylation and other clinicopathologic variables

Because there are many interfering factors in the KM survival curve, we need to further analyze by univariate and multivariate analysis. As shown in Table 3, univariate research gave out that methylation levels of CDKN2A gene was connected to better OS (P = 0.025976). Similarly, the methylation levels of cg03079681, cg04026675, cg07562918 and cg13601799 were also correlated with better OS (P = 0.002044, P = 0.001725, P = 0.038102 and P = 0.028328, sequentially). Other clinicopathologic parameters that were associated with poor survival included age, tumor invasion, lymph nodes involvement and advanced stage (P = 0.016085, P = 0.015629, P = 0.002664 and P = 0.000313, individually). Furthermore, in multivariate analysis, low methylation of CDKN2A gene, cg03079681, cg04026675, cg07562918 and cg13601799 locus associated with better OS (HR = 0.08, 95%CI: 0.01–0.82, P = 0.034; HR = 0.05, 95%CI: 0.00–0.46, P = 0.008; HR = 0.18, 95%CI: 0.06–0.56, P = 0.003; HR = 0.22, 95%CI: 0.05–0.99, P = 0.048; and HR = 0.37, 95%CI: 0.14–0.92, P = 0.033 respectively) (Figure (5a-f)).

**Table 3.** Univariate prognostic analysis for OS

Clinical factor	Univariate analysis	
	p-value	HR (95% CI)
Age (Years)	0.02	1.02 (1.00-1.04)
Gender (Female vs. Male)	0.11	1.40 (0.93-2.12)
Grade (G1 vs. G2-G3)	0.97	0.97 (0.24-3.94)
Stage (I-II vs. III-IV)	0.06	1.93 (0.97-3.83)
Tumor (T1-2 vs. T3-4)	0.049	7.22 (1.01-51.79)
Lymph node involvement (N0 vs. N1-N3)	0.02	1.75 (1.11-2.75)
Metastasis (M0 vs. M1)	0.19	1.68 (0.78-3.62)
CDKN2A methylation (low vs. High)	0.03	0.09 (0.01-0.75)
cg03079681 methylation (low vs. high)	0.00	0.03 (0.00-0.29)
cg04026675 methylation (low vs. high)	0.00	0.18 (0.06-0.53)
cg07562918 methylation (low vs. high)	0.04	0.23 (0.06-0.92)
cg13601799 methylation (low vs. high)	0.03	0.37 (0.15-0.90)

HR, hazard ratio; CI, conﬁdence interval

## GSEA analysis in CDKN2A gene methylation

**Figure 6.**
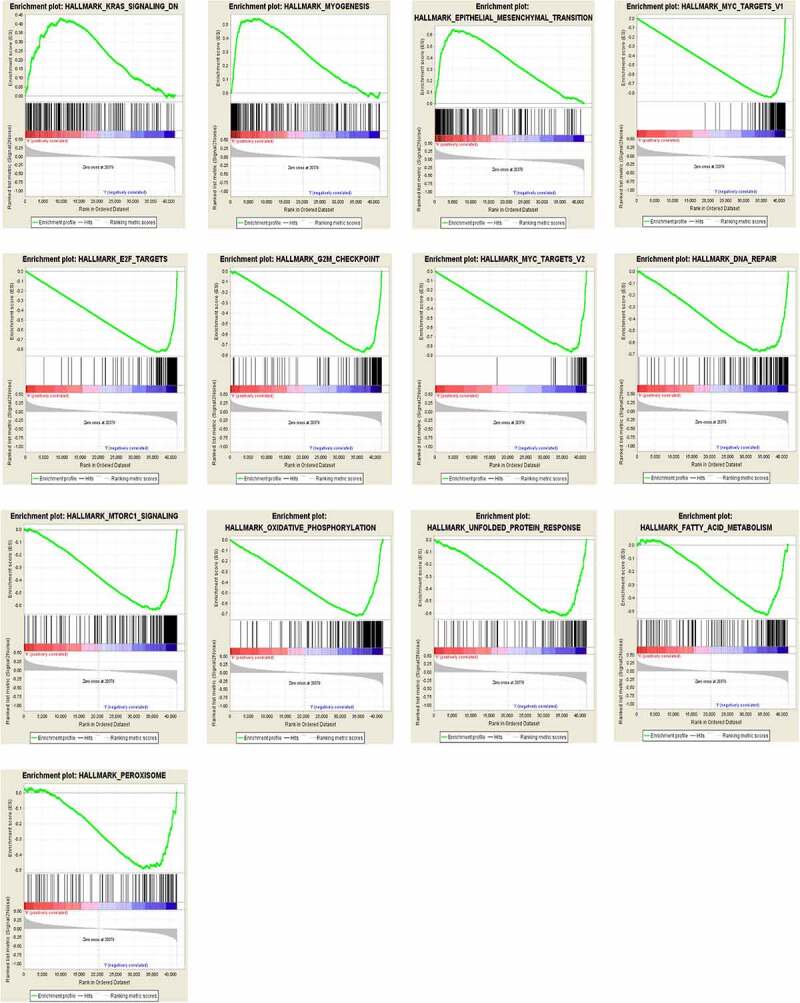
Panel with 3 gene sets enriched for CDKN2A gene hypermethylation and 10 gene sets enriched for CDKN2A gene hypomethylation in GC

To identify signaling pathways that might participate in the methylation processes, we conducted GSEA analysis and compared the results between low and high CDKN2A gene methylation samples in 304 specimens of GC. The GSEA analysis disclosed remarkable variation (FDR < 0.25, NOM p < 0.05) in improvement of MSigDB Collection (h.all.v7.0.symbols). Next, we choose the most considerably enhanced signaling pathways in regards to their normalized enrichment score (NES) (Table 4 and Figure 6). Our data suggested that Hallmark_kras_signaling_dn, Hallmark_myogenesis, and Hallmark_epithelial_mesenchymal_transition were uniquely supplemented in the CDKN2A gene hypermethylation phenotype. Furthermore, Hallmark_myc_targets_v1, Hallmark_e2f_targets, hallmark_g2m_checkpoint, Hallmark_myc_targets_v2, Hallmark_dna_repair, Hallmark_mtorc1_signaling, Hallmark_oxidative_phosphorylation, Hallmark_unfolded_protein_response, Hallmark_fatty_acid_metabolism and Hallmark_peroxisome were enriched in the CDKN2A gene hypomethylation phenotype. Notably, we hypothesised that aberrant methylation of CDKN2A gene is an important contributor in GC pathogenesis by regulating the above classic cancer-related signaling pathways.

**Table 4.** Enriched hallmark gene sets for CDKN2A gene methylation phenotype in GC

Phenotype	Hallmark Name	NES	NOM p- value	FDR q- value
CDKN2A hypermethylation	HALLMARK_KRAS_SIGNALING_DN	1.751036	0.002004	0.073121
	HALLMARK_MYOGENESIS	1.837245	0.019646	0.058606
	HALLMARK_EPITHELIAL_MESENCHYMAL_TRANSITION	1.886141	0.027027	0.060298
CDKN2A hypomethylation	HALLMARK_MYC_TARGETS_V1	-2.11244	0	0.008884
	HALLMARK_E2F_TARGETS	-2.07008	0	0.006287
	HALLMARK_G2M_CHECKPOINT	-2.04788	0	0.00521
	HALLMARK_MYC_TARGETS_V2	-2.01525	0	0.006721
	HALLMARK_DNA_REPAIR	-2.00303	0	0.006554
	HALLMARK_MTORC1_SIGNALING	-1.91897	0.005814	0.015038
	HALLMARK_OXIDATIVE_PHOSPHORYLATION	-1.94053	0.007678	0.014106
	HALLMARK_UNFOLDED_PROTEIN_RESPONSE	-1.8115	0.021526	0.038005
	HALLMARK_FATTY_ACID_METABOLISM	-1.73278	0.032946	0.063339
	HALLMARK_PEROXISOME	-1.59951	0.04142	0.119568

Gene sets with NOM P-value < 0.05 and FDR q-value < 0.25 were considered as signiﬁcantly enriched. NES, normalized enrichment score; NOM, nominal; FDR, false discovery rate.

## Immunohistochemical and methylation tissue examination

**Figure 7.**
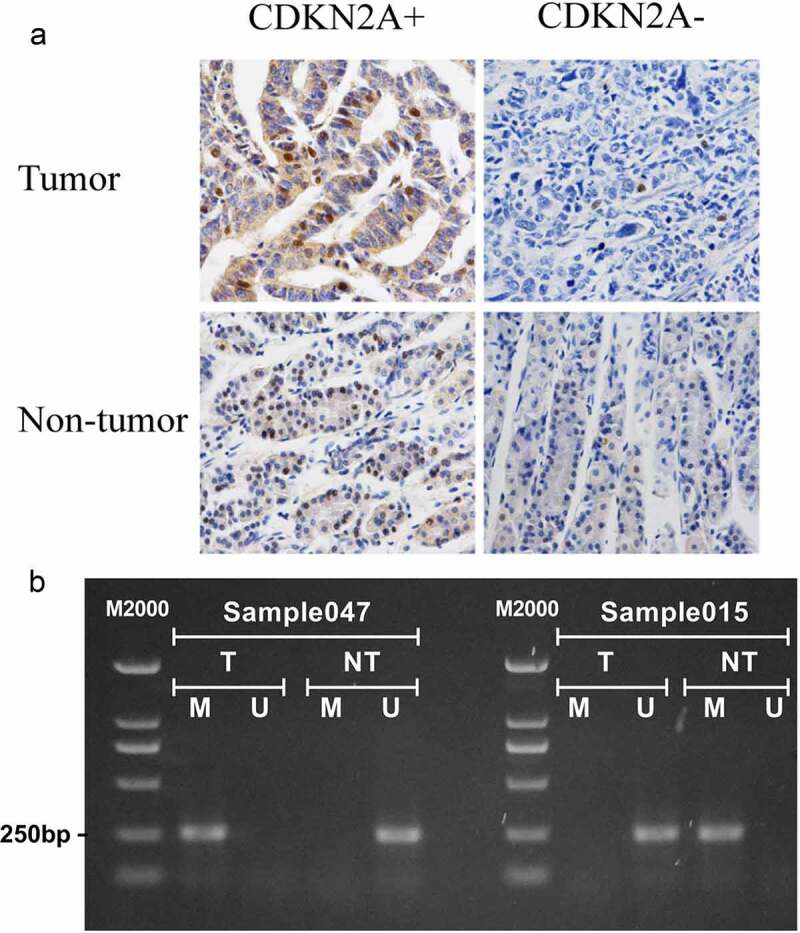
Immunohistochemistry and CDKN2A methylation-specific PCR in GC and adjacent non-tumor tissues. (a) Representative examples of IHC staining for CDKN2A protein expression (Magnification, 400×). (b) Representative examples of methylation analysis of CDKN2A gene promoter. U, unmethylation; M, methylation; T, primary gastric carcinoma tissues; NT, adjacent non-tumor tissues

To confirm if the results we observed in bioinformatics analyses successfully translated into clinic, we analyzed the expression of CDKN2A protein by IHC (Figure 7(a)) and the methylation by MS-PCR with 62 GC specimens and 62 non-tumor tissues (Figure 7(b)). Our data displayed a number of promising expressions of CDKN2A in cancer tissues that were strikingly inferior than that of the non-tumorous (P = 0.004). Methylation rate of CDKN2A was critically higher than non-tumorous tissues (P = 0.048) (Table 5). Similarly, to what we observed in bioinformatics analyses, the outcomes implied that CDKN2A methylation was negatively correlated with CDKN2A expression (c2 = 16.175,P = 0.001,r = 0.455) (Table 6).

**Table 5.** CDKN2A gene methylation and expression in 62 gastric cancer tissues and 62 non-tumor tissues

Tissue (n=124)	CDKN2A methylation			CDKN2A expression		
	M	U	*P* value	+	-	*P* value
Tumor (n=62)	23	39	0.048	34	28	0.004
Non-tumor (n=62)	13	49		49	13	

U, unmethylation; M, methylation.

**Table 6.** The association between methylation and gene expression in 62 tumor tissues

Tumor (n=62)	CDKN2A methylation		P value
	M (n=23)	U (n=39)	
CDKN2A expression			
+ (34)	5	29	0.001
- (28)	18	10	

U, unmethylation; M, methylation.

## Discussion

It has been known that aberrant DNA methylation is included in the pathogenicity of separate kinds of disease, especially cancer [12]. There are increasing evidence suggest that measuring DNA methylation is not only important for early disease diagnosis but can also help to evaluate treatment efficacy and estimate prognosis in GC patients [13,14].

Tumor suppressor genes located at CDKN2A/B locus (p15INK4b, p16INK4a, and p14ARF) are most regularly silenced or deleted in cancers relating to humans. DNA methylation decreases p16INK4a expression and eventually relieves the checkpoint for oncogene-encouraged aging [15]. Nevertheless, the aberrant expression of CDKN2A (p16) is frequently observed in human cancers. Indeed, the up-regulated expression of CDKN2A (p16) in sebaceous carcinoma [16] and colon cancer [17] has been exhibited. On the other hand, decreased expression of CDKN2A might play an important role in head and neck squamous cell carcinoma (HNSCC) that present with negative HPV results [18]. Furthermore, the methylated exon 2 of CDKN2A gene was considerably excessive when compared with CDKN2A promoter in breast carcinoma, and therefore, could have a connection to breast carcinogenesis [19]. CDKN2A gene exon 2 was also found to be methylated in esophageal tumors [20].

In this study, database analysis of TCGA and GSE30601 illustrated that methylation levels of CDKN2A gene were outstandingly elevated in GC compared with the normal tissues. Similarly, we have confirmed in clinical samples that methylation locus was more frequent in GC tissues than in para cancer non-tumor tissues (P < 0.05). Our findings were consistent with a previous meta-analysis based on Chinese patients which showed that the average methylation rate for GC sat at 43.3% with a scale of 28.3–64.4%, while the mean rate of methylation for controlled (i.e. the healthy) was 0.0% with a field of 0.0–13.3% [21]. Our data suggested that the increasing methylation rate of CDKN2A gene in GC was associated with advanced age (P = 9.083e-04), and the rate of methylation of cg00718440 was notably increased in males in comparison to female patients (P = 0.015). Furthermore, the methylation of cg12840719 was significantly more common in patients with G2 grade (P = 0.004). These results suggested that the methylation of different locus may exert different effects in the pathogenesis of GC and more studies are needed to better understand the differences.

Previous studies showed that neither CDKN2A gene nor CDKN2A (p16) protein expression was an independent predictor, but the combination of gene and protein status was an important predictor of urothelial bladder carcinoma [22]. There were studies also showing the presence of both CDKN2A (p16) overexpression and hypermethylated epigenetic changes in CDKN2A gene promoters in periocular sebaceous adenocarcinoma [23]. There has been knowledge stating that hypermethylation of the CDKN2A gene can induce erosion of CDKN2A expression in many malignancies, involving hepatocellular carcinoma [24], cervical cancer [25], and non-small cell lung cancer [26]. Similarly, in this study, the data gained illustrated that the expression of CDKN2A was in negative correlations with the methylation level of CDKN2A gene and several loci (cg00718440, cg03079681, cg04026675, cg07562918, cg10848754, cg14069088, and cg14430974). However, the methylation level of cg12840719 was in direct correlations with CDKN2A gene expression (rs = 0.483, P = 2.667e-19). This discrepancy between locus cg12840719 and other loci is interesting and may help to better understand the regulation of CDKN2A gene expression.

It has been known that CDKN2A gene hypermethylation is in correspondence with a shorter OS and repetition-free survival time in HNSCC [27]. However, such association was not observed in colorectal cancer but patients with CDKN2A gene promoter methylation and/or loss of CDKN2A (p16) were connected to a decrease in OS [28]. There was no consensus on the prognostic value of CDKN2A gene methylation in primary GC. In our study, bioinformatics analysis showed that CDKN2A gene methylation was a good prognostic predictor for primary GC. Multivariate analysis suggested that CDKN2A gene methylation was individually related to OS (P = 0.004). Similarly, the methylation of cg03079681, cg04026675, cg07562918, and cg13601799 were also associated with good OS. However, the expression and of CDKN2A gene were not remarkably tied in with patient survival.

Further investigation of possible downstream targets and malignancy coupled signaling routes for CDKN2A gene hypermethylation. In this study, we divided TCGA GC samples in two according to the mean CDKN2A gene methylation level before we evaluated the enrichments by GSEA. GSEA results showed that Hallmark_kras_signaling_dn, Hallmark_myogenesis, and Hallmark_epithelial_mesenchymal_transition were differentially enriched in samples with CDKN2A gene hypermethylation. Furthermore, Hallmark_myc_targets_v1, Hallmark_e2f_targets, Hallmark_g2m_checkpoint, Hallmark_myc_targets_v2, Hallmark_dna_repair, Hallmark_mtorc1_signaling, Hallmark_oxidative_phosphorylation, Hallmark_unfolded_protein_response, Hallmark_fatty_acid_metabolism, and Hallmark_peroxisome were differentially enriched in samples with CDKN2A gene hypomethylation. Notably, the data gained supported the fact both hypermethylation and hypomethylation of the CDKN2A gene region played a vital role in tumor genesis by regulating classic cancer-related signaling pathways. CDKN2A gene aberrant methylation might serve as a potential prognostic marker, as well as a potential therapeutic target in GC.

There were some limitations in our study. Firstly, we only detected the methylation of the region of CDKN2A gene in GC tissues, instead of using the Methylation 450 K Beadchip. Secondly, the expression of CDKN2A in GC is affected by a variety of regulatory mechanisms except for gene methylation and the result might be biased by some potentially confounding factors.

## Conclusions

In conclusion, the aberrant methylation of CDKN2A gene and its special locus region may be a predictor for better prognosis in GC. Moreover, the hypermethylation of CDKN2A may participate in the pathogenesis of GC associated with the down-regulation of CDKN2A gene expression and Hallmark_kras_signaling_dn, Hallmark_myogenesis, and Hallmark_epithelial_mesenchymal_transition pathways. Further studies should be performed to verify the bioinformatics results and discover the biologic impact of these findings.

## Highlights

1. CDKN2A gene methylation was increased in GC tissues.
2. The level of CKDN2A methylation locus were elevated in GC tissues.
3. Low methylation of CDKN2A, cg03079681, cg04026675, cg07562918, and cg13601799 locus indicated a better prognosis in GC.
